# Prediction of short-term atrial fibrillation risk using primary care electronic health records

**DOI:** 10.1136/heartjnl-2022-322076

**Published:** 2023-02-09

**Authors:** Ramesh Nadarajah, Jianhua Wu, David Hogg, Keerthenan Raveendra, Yoko M Nakao, Kazuhiro Nakao, Ronen Arbel, Moti Haim, Doron Zahger, John Parry, Chris Bates, Campbel Cowan, Chris P Gale

**Affiliations:** 1 Leeds Institute for Data Analytics, University of Leeds, Leeds, UK; 2 Leeds Institute of Cardiovascular and Metabolic Medicine, University of Leeds, Leeds, UK; 3 Department of Dentistry, University of Leeds, Leeds, UK; 4 School of Computing, University of Leeds, Leeds, UK; 5 Faculty of Medicine and Health, University of Leeds, Leeds, UK; 6 Maximizing Health Outcomes Research Lab, Sapir College, Hof Ashkelon, Israel; 7 Community Medical Services Division, Clalit Health Services, Tel Aviv, Israel; 8 Department of Cardiology, Soroka University Medical Center, Beer Sheva, Israel; 9 Faculty of Health Sciences, Ben-Gurion University of the Negev, Beer Sheva, Israel; 10 Cardiology, Soroka Medical Center, Beer Sheva, Israel; 11 The Phoenix Partnership, Leeds, UK; 12 Cardiology, Leeds General Infirmary, Leeds, UK

**Keywords:** biostatistics, atrial fibrillation, electronic health records

## Abstract

**Objective:**

Atrial fibrillation (AF) screening by age achieves a low yield and misses younger individuals. We aimed to develop an algorithm in nationwide routinely collected primary care data to predict the risk of incident AF within 6 months (Future Innovations in Novel Detection of Atrial Fibrillation (FIND-AF)).

**Methods:**

We used primary care electronic health record data from individuals aged ≥30 years without known AF in the UK Clinical Practice Research Datalink-GOLD dataset between 2 January 1998 and 30 November 2018, randomly divided into training (80%) and testing (20%) datasets. We trained a random forest classifier using age, sex, ethnicity and comorbidities. Prediction performance was evaluated in the testing dataset with internal bootstrap validation with 200 samples, and compared against the CHA_2_DS_2_-VASc (Congestive heart failure, Hypertension, Age >75 (2 points), Stroke/transient ischaemic attack/thromboembolism (2 points), Vascular disease, Age 65–74, Sex category) and C_2_HEST (Coronary artery disease/Chronic obstructive pulmonary disease (1 point each), Hypertension, Elderly (age ≥75, 2 points), Systolic heart failure, Thyroid disease (hyperthyroidism)) scores. Cox proportional hazard models with competing risk of death were fit for incident longer-term AF between higher and lower FIND-AF-predicted risk.

**Results:**

Of 2 081 139 individuals in the cohort, 7386 developed AF within 6 months. FIND-AF could be applied to all records. In the testing dataset (n=416 228), discrimination performance was strongest for FIND-AF (area under the receiver operating characteristic curve 0.824, 95% CI 0.814 to 0.834) compared with CHA_2_DS_2_-VASc (0.784, 0.773 to 0.794) and C_2_HEST (0.757, 0.744 to 0.770), and robust by sex and ethnic group. The higher predicted risk cohort, compared with lower predicted risk, had a 20-fold higher 6-month incidence rate for AF and higher long-term hazard for AF (HR 8.75, 95% CI 8.44 to 9.06).

**Conclusions:**

FIND-AF, a machine learning algorithm applicable at scale in routinely collected primary care data, identifies people at higher risk of short-term AF.

**WHAT IS ALREADY KNOWN ON THIS TOPIC**

- European Society of Cardiology Guidelines recommend opportunistic screening in individuals aged ≥65 years and systematic screening in individuals aged ≥75 years. However, this approach achieves low yields and misses the increasing number of people diagnosed with atrial fibrillation (AF) before the age of 65 years.
- Several AF risk prediction algorithms have been tested using community-based electronic health records (EHRs). However, current models are limited by moderate discrimination performance, limited scalability and long prediction horizons, which are not relevant to the decision to investigate for AF in the short term.

**WHAT THIS STUDY ADDS**

- In this nationwide primary care EHR study, we show that a random forest classifier (Future Innovations in Novel Detection of Atrial Fibrillation (FIND-AF)) can be used to accurately predict AF risk within 6 months, superior to the C_2_HEST and CHA_2_DS_2_-VASc scores, and can be applied to all UK primary care EHRs.
- One-fifth of incident AF cases in 6 months occurred in individuals younger than 65 years who would ordinarily be excluded from AF screening programmes. FIND-AF identified a cohort of higher-risk individuals younger than 65 years of age, and higher predicted AF risk was associated with elevated incident AF in the short and long term.

**HOW THIS STUDY MIGHT AFFECT RESEARCH, PRACTICE OR POLICY**

- Leveraging FIND-AF, a scalable machine learning algorithm, in routinely collected EHRs may improve the efficiency of diagnostic pathways for AF.
- External validation and evaluation of prospective clinical deployment of FIND-AF are in process, and a cost utility analysis and budget impact analysis will need to be conducted.

## Introduction

Atrial fibrillation (AF) is a major public health issue. There are now more new cases of AF diagnosed each year in the English National Health Service (NHS) than the four most common causes of cancer combined.^1^ Moreover, it is estimated that up to 35% of disease burden remains undiagnosed,^2^ and 15% of strokes occur in the context of undiagnosed AF.^3^

Early detection of AF may permit the initiation of oral anticoagulation to reduce embolic stroke risk,^4^ and early antiarrhythmic therapy to reduce the risk of death and stroke.^5^ Accordingly, early AF detection is a key cardiovascular priority in the UK NHS Long Term Plan,^6^ and the European Society of Cardiology recommends opportunistic screening by pulse palpation or ECG rhythm strip in persons aged ≥65 years and systematic ECG screening in those aged ≥75 years.^7^ However, there is an increasing cohort of individuals aged younger than 65 years who are being diagnosed with AF and are eligible for anticoagulation.^1^

A large proportion of the population is registered in primary care with a routinely collected electronic health record (EHR).^8 9^ An algorithm that uses routinely collected EHR data to calculate AF risk could give a scalable, efficient and fair approach to targeting AF detection. However, previous algorithms tested in community-based EHRs have a number of shortcomings (online supplemental tables 1 and 2). First, many algorithms developed using traditional regression techniques show only moderate discriminative performance.^10^ Second, algorithm prediction horizons are often 5 or 10 years, making it difficult to judge the merits of investigating individuals in the short term.^9 11^ Third, reports have infrequently investigated for variation in algorithm prediction performance by sex and ethnicity.^11^ Fourth, algorithms often require variables frequently missing from routinely collected data such as height, weight and blood pressure thereby restricting the population to which they can be applied.^9 11^

10.1136/heartjnl-2022-322076.supp1 Supplementary data

Therefore, our objective was to train and test an algorithm (Future Innovations in Novel Detection of Atrial Fibrillation, FIND-AF) that predicts an individual’s risk of AF in the next 6 months using routinely recorded data in primary care EHRs. We compared performance against other AF prediction algorithms and investigated for variation in performance by sex and ethnicity.

## Methods

### Study design and population

In this population-based study, we used primary care EHRs from the UK Clinical Practice Research Datalink (CPRD)-GOLD dataset. CPRD is one of the largest databases of longitudinal medical records from primary care worldwide and contains anonymised patient data from approximately 7% of the UK population.^8^ CPRD-GOLD represents the UK population in terms of age, sex and ethnicity,^8^ and has been used to develop algorithms for predicting AF.^11^ Data collection happens as part of routine clinical care in participating practices and patients are included in the primary care dataset from their first until their last contact with a participating practice.^8^ Diagnostic coding for AF in CPRD has been shown to be consistent and valid, with a positive predictive value (PPV) of 98%.^12^

All individuals in the CPRD dataset were linked to Hospital Episode Statistics (HES) Admitted Patient Care (APC) records to obtain comprehensive coverage of AF cases diagnosed in secondary care. We included all adults registered at practices within CPRD who were ≥30 years of age at entry with no history of AF from either data source and at least 1-year follow-up between 2 January 1998 and 30 November 2018. Individuals were censored to a diagnosis of AF (or atrial flutter (AFl), since it has similar thromboembolic risk and anticoagulation guidelines),^7^ withdrawal from CPRD or 6 months, whichever came first. Diagnoses of AF or AFl in primary care were identified using Read codes in CPRD and in secondary care with the 10th revision of the International Statistical Classification of Diseases and Related Health Problems codes in HES-APC (online supplemental table 3). Individuals were randomly split 4:1 to establish a training dataset (80%) and a testing dataset (20%) using the Mersenne twister pseudorandom number generator.

We followed the Transparent Reporting of a Multivariable Prediction Model for Individual Prognosis or Diagnosis reporting guideline and the CODE-EHR best-practice framework for using structured electronic healthcare records in clinical research.^13 14^

### FIND-AF algorithm development

A random forest (RF) classifier was trained to predict AF at 6 months. Our systematic review evidenced strong discriminative performance for AF prediction using RF across different EHR datasets.^10^ RF is a machine learning method consisting of many individual decision trees that operate as an ensemble.^15^ FIND-AF was trained using 10-fold cross-validation on the full training set (full details available in online supplemental methods).

To create an algorithm that could be implemented at scale in national primary care EHRs, we restricted candidate variables to age, sex, comorbidities (72 binary variables, indicating presence or absence of recorded diagnosis) and ethnicity (six categories; online supplemental table 6). Observations and laboratory results were not included. Ethnicity information is routinely collected in the UK NHS and so has increasingly high completeness,^16^ and we included an ‘ethnicity unrecorded’ category where it was unavailable because missingness was considered to be informative.^17^ Predictor variables were selected a priori from systematic review of variables included in previous AF risk prediction algorithms,^10^ plus an updated literature review (online supplemental tables 4–6). Diagnostic code lists only included the primary care coding system (Read codes), ensuring that only information readily available within a primary care EHR could be incorporated within the algorithm. Concordantly, our entire analytical cohort had no missing data for any of the predictor variables and the algorithm could be applied to all records.

### Statistical analyses

The baseline characteristics are summarised by incident AF status. Continuous variables were reported as mean±SD. Categorical variables were reported as frequencies with corresponding percentages.

The degree of variation of each feature in FIND-AF to classification was calculated using the mean decrease in the Gini coefficient, a measure of how each variable contributes to the homogeneity of nodes and leaves in the resulting RF.

Model performance of FIND-AF was determined using the full holdout test set with internal bootstrap validation with 200 samples and compared with a multivariable logistic regression (MLR) model developed with backward model selection with Akaike information criterion.^18^ Performance was compared with the CHA_2_DS_2_-VASc (Congestive heart failure, Hypertension, Age >75 (2 points), Stroke/transient ischaemic attack/thromboembolism (2 points), Vascular disease, Age 65–74, Sex category) and C_2_HEST (Coronary artery disease/Chronic obstructive pulmonary disease (1 point each), Hypertension, Elderly (age ≥75, 2 points), Systolic heart failure, Thyroid disease (hyperthyroidism)) scores. The CHA_2_DS_2_-VASc score was originally developed to predict stroke risk in individuals with AF, and the C_2_HEST score for Asian people without structural heart disease.^10^ These algorithms are robust to missing data in routinely collected primary care EHRs and have been tested for AF risk prediction in European cohorts (online supplemental table 2).^10^ Other algorithms that can only be applied to a minority of European primary care EHRs (Pfizer-AI, CHARGE-AF) were not considered.^9 19^ The area under the receiver operating characteristic (AUROC) curve was used to evaluate predictive ability (concordance index) with 95% CIs calculated using the DeLong method. Youden Index was established for the outcome measure as a method of empirically identifying the optimal dichotomous cut-off to assess sensitivity, specificity, PPV and negative predictive value (NPV). Youden Index was calculated and optimised for each test set for each score to derive the optimal cut-off threshold. Calibration was assessed by plotting predicted AF risk against observed AF incidence and by the calibration slope. We calculated the Brier score, a measure of both discrimination and calibration, by taking the mean squared difference between predicted probabilities and the observed outcome. To assess the clinical impact of using FIND-AF as opposed to other risk prediction scores, we calculated the net reclassification index at 0.4% AF risk threshold (the average 6-month incidence rate in the cohort) and conducted a decision curve analysis.

We investigated the performance of FIND-AF, CHA_2_DS_2_-VASc and C_2_HEST within relevant subgroups defined by sex, ethnicity (white vs black vs Asian vs other non-white ethnic minorities) and age (≥65 years and ≥75 years). We plotted Kaplan-Meier plots for individuals identified as higher and lower FIND-AF-predicted risk of AF to assess the event rate for AF censored at 10 years, and calculated the HR for AF between higher and lower FIND-AF-predicted risk of AF using the Cox proportional hazard model with adjustment for the competing risk of death. We used R V.4.1.0 for all analyses.

### Patient and public involvement

The Arrhythmia Alliance, an AF association, provided input on the FIND-AF scientific advisory board. The FIND-AF patient and public involvement group have given input to reporting and dissemination plans of the research.

## Results

### Patient population

There were 2 081 139 individuals registered in our UK primary care cohort (1 664 911 in the training dataset, 416 228 in testing dataset), with average age 49.9 years (SD 15.4), 50.7% women and 86.7% white. Baseline characteristics and clinical outcomes were similar in the training and testing datasets (online supplemental table 7). Within 6 months, 7386 individuals (0.4%) were recorded as having AF. Those who developed AF were older and had a higher prevalence of baseline comorbidities than individuals who did not develop AF (table 1). Of new cases, 1546 (20.9%) were younger than 65 years old.

**Table 1:** Baseline characteristics of analytical cohort with and without atrial fibrillation (AF)

	Incident AF	
	No AF n (%)	AF n (%)
	2 073 753	7386
Demographics		
Age, years	49.82 (15.37)	73.72 (12.62)
Sex (women)	1 051 942 (50.7)	3619 (49.0)
Comorbidities		
Diabetes mellitus	71 966 (3.5)	815 (11.0)
Stroke or TIA	37 773 (1.8)	892 (12.1)
Ischaemic heart disease	77 060 (3.7)	1542 (20.9)
Hypertension	247 436 (11.9)	2887 (39.1)
Heart failure	13 717 (0.7)	650 (8.8)
Dyslipidaemia	60 357 (2.9)	532 (7.2)
Hyperthyroidism	16 147 (0.8)	155 (2.1)
COPD	24 962 (1.2)	461 (6.2)
Chronic kidney disease	29 359 (1.4)	449 (6.1)
Anaemia	66 844 (3.2)	501 (6.8)
Cancer	72 621 (3.5)	887 (12.0)
Valvular heart disease	9 497 (0.5)	376 (5.1)
Mean CHA_2_DS_2_-VASc score (SD)	0.97 (1.03)	2.72 (1.42)

CHA_2_DS_2_-VASc, Congestive heart failure, Hypertension, Age >75 years (2 points), Stroke/transient ischaemic attack/thromboembolism (2 points), Vascular disease, Age 65–74 years, Sex category; COPD, chronic obstructive pulmonary disease; TIA, transient ischaemic attack.

### Prediction factors and model accuracy

**Figure 1:**
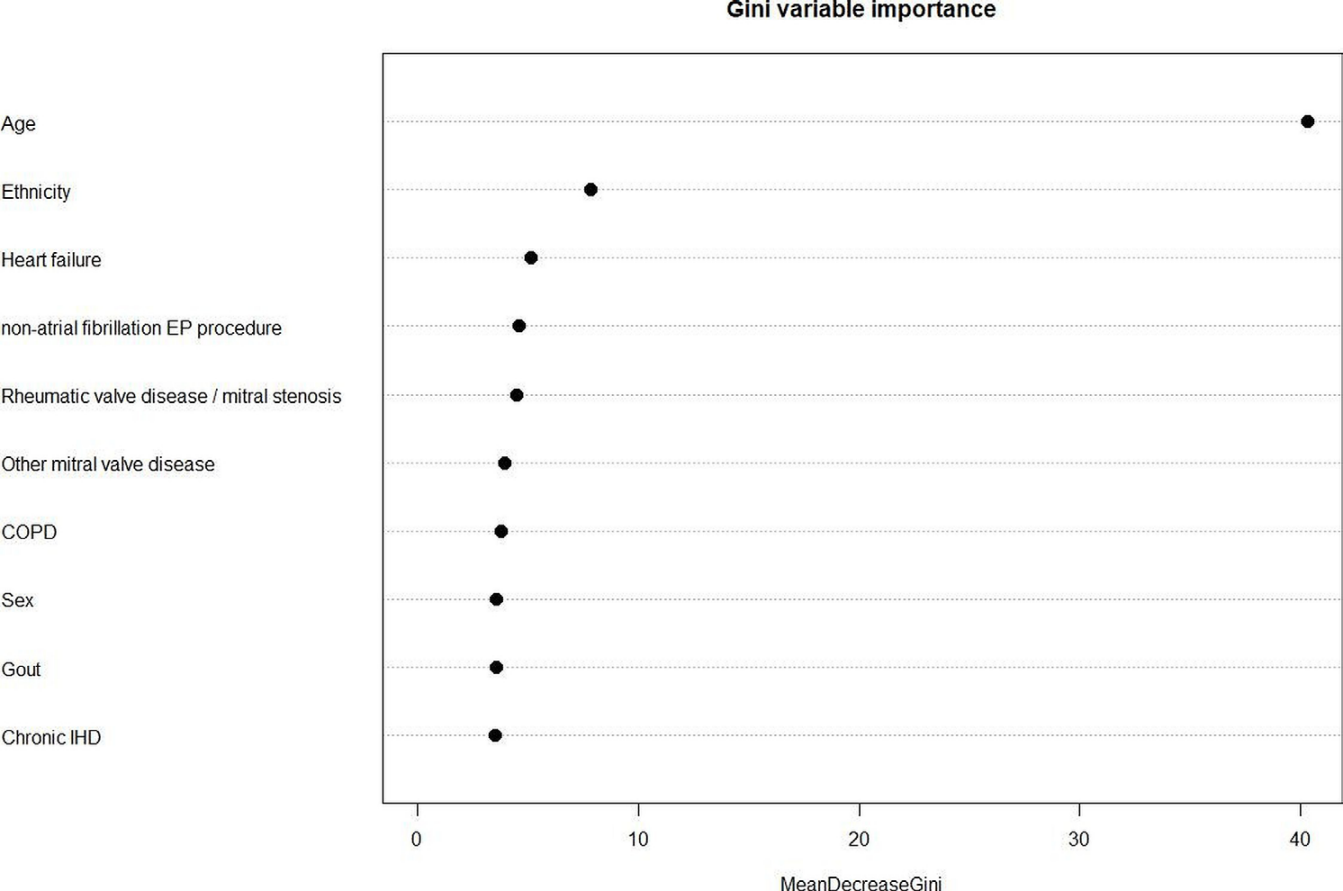
The top 10 most important variables for FIND-AF prediction in individuals aged ≥30 years quantified by mean decrease in Gini coefficient. COPD, chronic obstructive pulmonary disease; EP, electrophysiology; FIND-AF, Future Innovations in Novel Detection of Atrial Fibrillation; IHF, ischaemic heart disease.

**Figure 2:**
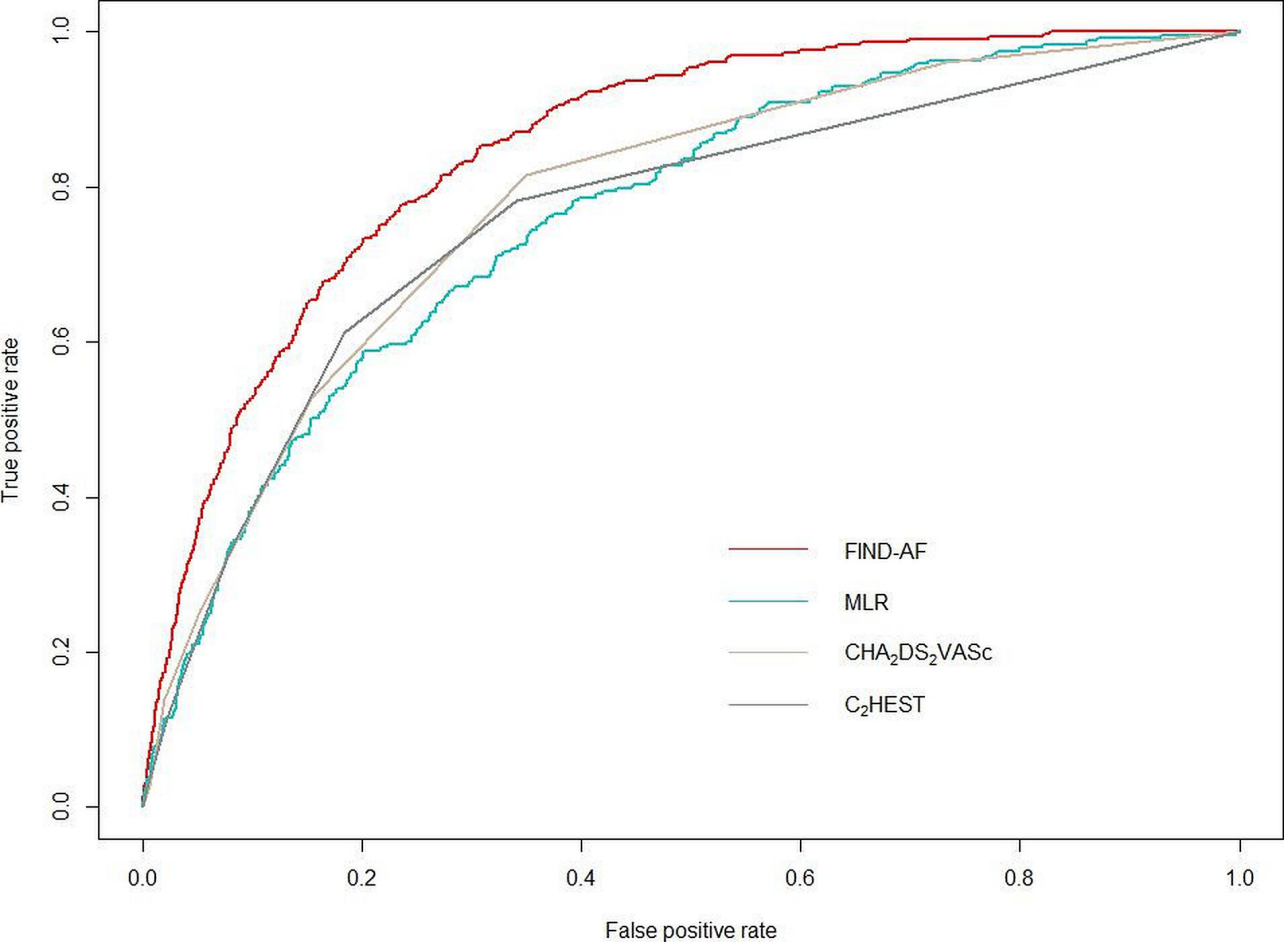
Receiver operating characteristic curves for FIND-AF, the multivariable logistic regression (MLR), CHA_2_DS_2_-VASc and C_2_HEST algorithm. C_2_HEST, Coronary artery disease/Chronic obstructive pulmonary disease (1 point each), Hypertension, Elderly (age ≥75, 2 points), Systolic heart failure, Thyroid disease (hyperthyroidism); CHA_2_DS_2_-VASc, Congestive heart failure, Hypertension, Age >75 (2 points), Stroke/transient ischaemic attack/thromboembolism (2 points), Vascular disease, Age 65–74, Sex category.

According to mean decrease in the Gini coefficient, age contributed the most to the prediction, followed by ethnicity and history of heart failure (figure 1). AF discrimination and accuracy of predictions, by AUROC and Brier scores, were better using FIND-AF than the MLR, CHA_2_DS_2_-VASc and C_2_HEST algorithms (table 2 and figure 2). Sensitivity was highest for the CHA_2_DS_2_-VASc algorithm, but specificity lowest.

**Table 2:** Performance for 6-month incident AF with optimal threshold determined by Youden Index

	Algorithm			
	FIND-AF	MLR	CHA_2_DS_2_-VASc	C_2_HEST
AUROC (95% CI)	0.824 (0.814 to 0.834)	0.765 (0.755 to 0.769)	0.784 (0.773 to 0.794)	0.757 (0.744 to 0.770)
Sensitivity (95% CI)	0.781 (0.731 to 0.829)	0.760 (0.653 to 0.814)	0.847 (0.829 to 0.866)	0.642 (0.619 to 0.791)
Specificity (95% CI)	0.731 (0.693 to 0.771)	0.679 (0.635 to 0.776)	0.611 (0.608 to 0.612)	0.790 (0.622 to 0.792)
PPV (%(95% CI))	2.5% (2.3 to 2.7)	2.0% (1.8 to 2.6)	2.2% (2.1 to 2.3)	2.0% (1.5 to 2.2)
NPV (%(95% CI))	99.8% (99.8 to 99.8)	99.7% (99.6 to 99.7)	99.8% (99.8 to 99.8)	99.7% (99.7 to 99.8)
Calibration slope* (95% CI)	0.782 (0.743 to 0.824)	0.698 (0.654 to 0.735)	0.621 (0.589 to 0.652)	0.608 (0.576 to 0.648)
Brier score	0.069	0.097	0.093	0.102

*Calibration slope was derived from linear regression models by forcing the intercept through origin (0, 0).

AF, atrial fibrillation; AUROC, area under the receiver operating characteristic; CHA_2_DS_2_-VASc, Congestive heart failure, Hypertension, Age >75 (2 points), Stroke/transient ischaemic attack/thromboembolism (2 points), Vascular disease, Age 65–74, Sex category; C_2_HEST, Coronary artery disease/Chronic obstructive pulmonary disease (1 point each), Hypertension, Elderly (age ≥75, 2 points), Systolic heart failure, Thyroid disease (hyperthyroidism); FIND-AF, Future Innovations in Novel Detection of Atrial Fibrillation; MLR, multivariable logistic regression; NPV, negative predictive value; PPV, positive predictive value.

According to the Youden Index, the optimal cut-off was 0.0032, leading to a sensitivity of 78% and a specificity of 73%, with a PPV of 2.5% and NPV of 99.8%. The low incidence of AF over 6 months led to similar values for PPV and NPV across the algorithms. Of the algorithms, FIND-AF was the best calibrated (calibration slope 0.782 (95% CI 0.743 to 0.824), table 2 and online supplemental figure 1), yet showed underestimation of risk in the mid-risk strata and overestimation in the highest risk strata.

### Risk classification

Of the 416 228 individuals in the testing set, 82 942 (19.9%) were classified as higher risk using FIND-AF, 84 282 (20.2%) using the CHA_2_DS_2_-VASc score and 84 542 (20.3%) using the C_2_HEST score, respectively. Net reclassification analyses at the 0.4% risk threshold demonstrated modestly favourable reclassification using FIND-AF as opposed to using CHA_2_DS_2_-VASc (net reclassification 0.032, 95% CI 0.029 to 0.051) and strong favourable reclassification using FIND-AF as opposed to using C_2_HEST (net reclassification 0.113, 95% CI 0.098 to 0.135; online supplemental table 8). In a decision curve analysis, FIND-AF had a superior net benefit compared with the CHA_2_DS_2_-VASc and C_2_HEST risk scores across all threshold probabilities (online supplemental figure 2).

Of the 82 942 individuals identified as higher risk by FIND-AF, 3483 were <65 years of age, of whom 3448 had a CHA_2_DS_2_-VASc score of at least 1. The incidence rate of AF in routine clinical practice at 6 months was 20-fold higher among individuals identified as a higher predicted risk of AF by FIND-AF compared with individuals identified as lower risk (2.0% vs 0.1%). In routine clinical practice, 1 in every 71 individuals aged ≥65 years were diagnosed with AF within 6 months, 1 in every 58 individuals aged ≥75 years and 1 in every 40 individuals identified at higher predicted AF risk.

**Figure 3:**
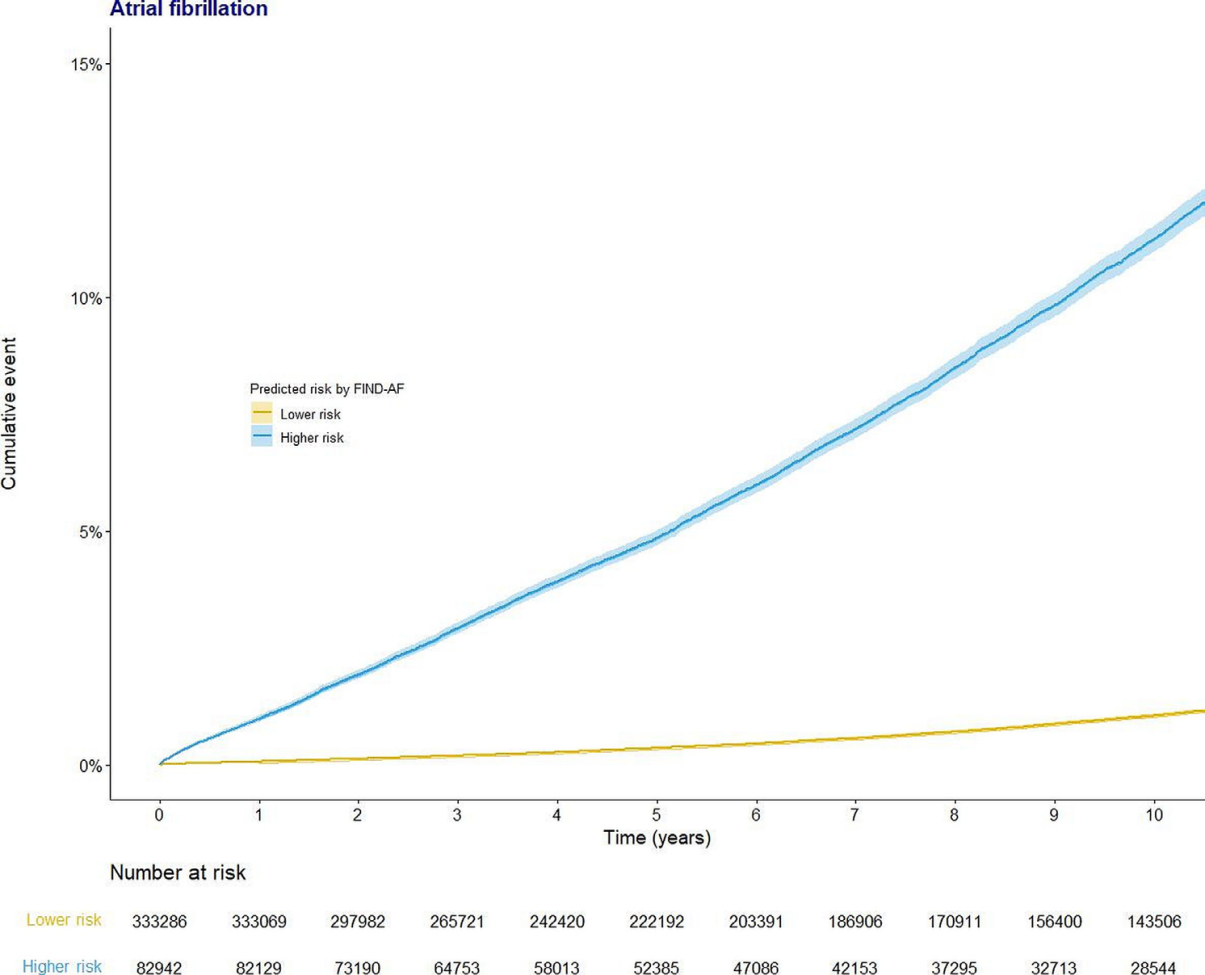
Kaplan-Meier plots for AF occurrence, by predicted risk from FIND-AF. AF, atrial fibrillation; FIND-AF, Future Innovations in Novel Detection of Atrial Fibrillation.

Higher predicted AF risk was also associated with increased long-term AF occurrence. Within 5 and 10 years, respectively, 5.1% and 11.9% of the higher predicted risk cohort had been diagnosed with AF, with an 8.75-fold increased hazard (95% CI 8.44 to 9.06) relative to individuals at lower predicted risk (figure 3).

### Model performance in clinically relevant subgroups

FIND-AF discrimination performance remained strong in both sexes, whereas for the CHA_2_DS_2_-VASc and C_2_HEST scores, performance was better in men than women (table 3). The scores performed differently across ethnic groups. In black individuals, AF discrimination was highest for CHA_2_DS_2_-VASc, and in white and Asian individuals, FIND-AF had the strongest discrimination performance.

**Table 3:** Discrimination performance of FIND-AF, CHA_2_DS_2_-VASc and C_2_HEST by sex, age and ethnicity

	FIND-AF	CHA_2_DS_2_-VASc	C_2_HEST
	AUROC (95% CI)	AUROC (95% CI)	AUROC (95% CI)
Overall	0.824 (0.814 to 0.834)	0.784 (0.773 to 0.794)	0.757 (0.744 to 0.770)
Sex			
Men	0.819 (0.809 to 0.829)	0.807 (0.793 to 0.821)	0.793 (0.777 to 0.810)
Women	0.821 (0.810 to 0.831)	0.776 (0.760 to 0.793)	0.746 (0.727 to 0.765)
Age			
≥65 years	0.712 (0.698 to 0.727)	0.669 (0.654 to 0.684)	0.675 (0.661 to 0.690)
≥75 years	0.657 (0.638 to 0.675)	0.612 (0.593 to 0.632)	0.589 (0.570 to 0.608)
Ethnicity			
White	0.810 (0.799 to 0.821)	0.781 (0.769 to 0.792)	0.756 (0.743 to 0.770)
Asian	0.796 (0.693 to 0.899)	0.758 (0.639 to 0.876)	0.731 (0.611 to 0.850)
Black	0.801 (0.680 to 0.923)	0.843 (0.764 to 0.923)	0.707 (0.511 to 0.902)
Other non-white ethnic minority	0.805 (0.765 to 0.845)	0.768 (0.729 to 0.807)	0.805 (0.765 to 0.846)
Ethnicity unrecorded	0.823 (0.770 to 0.875)	0.838 (0.777 to 0.900)	0.788 (0.705 to 0.870)

The total number of individuals in each subgroup and number of incident AF cases are as follows: men (n=211 378, AF=720), women (n=204 850, AF=753), age ≥65 years (n=81 258, AF=1168), age ≥75 years (n=36 358, AF=796), white (n=279 027, AF=1301), Asian (n=8422, AF=16), black (n=6478, AF=11), other non-white ethnic minority (n=28 303, AF=96), ethnicity unrecorded (n=93 998, AF=49).

AF, atrial fibrillation; AUROC, area under the receiver operating characteristic; CHA_2_DS_2_-VASc, Congestive heart failure, Hypertension, Age >75 (2 points), Stroke/transient ischaemic attack/thromboembolism (2 points), Vascular disease, Age 65–74, Sex category; C_2_HEST, Coronary artery disease/Chronic obstructive pulmonary disease (1 point each), Hypertension, Elderly (age ≥75, 2 points), Systolic heart failure, Thyroid disease (hyperthyroidism); FIND-AF, Future Innovations in Novel Detection of Atrial Fibrillation.

## Discussion

**Figure 4:**
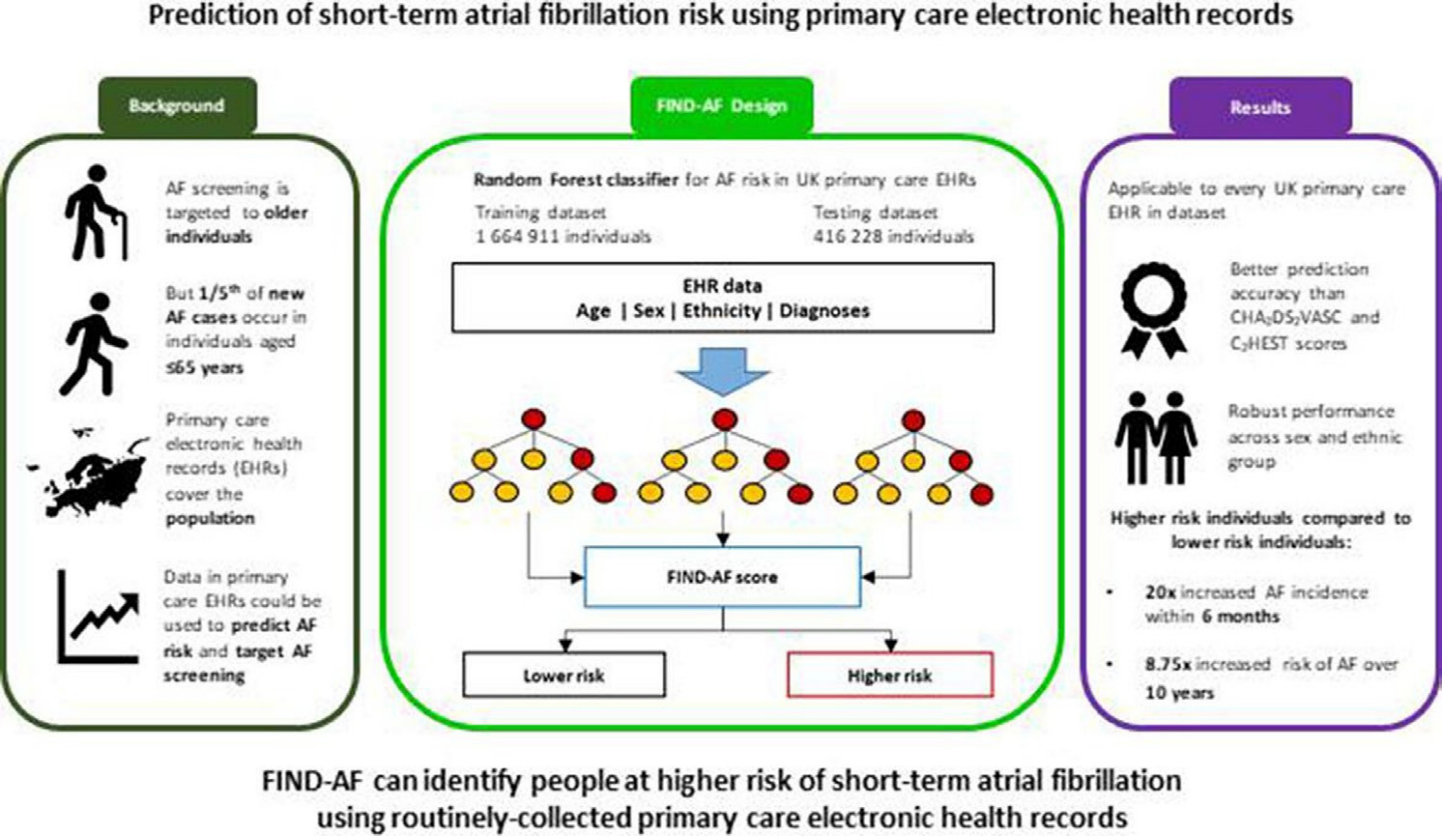
Summary of the study and main findings. Hitherto implementation of screening for atrial fibrillation (AF) has been targeted to older persons in the general population, but this may miss one-fifth of new cases. A machine learning algorithm using routinely collected data in primary care electronic health records in the UK can accurately predict short-term risk of AF in persons aged ≥30 years. This may be a more efficient method for guiding AF screening. C_2_HEST, Coronary artery disease/Chronic obstructive pulmonary disease (1 point each), Hypertension, Elderly (age ≥75, 2 points), Systolic heart failure, Thyroid disease (hyperthyroidism); CHA_2_DS_2_-VASc, Congestive heart failure, Hypertension, Age >75 (2 points), Stroke/transient ischaemic attack/thromboembolism (2 points), Vascular disease, Age 65–74, Sex category; FIND-AF, Future Innovations in Novel Detection of Atrial Fibrillation.

In this population-based study, we trained a machine learning algorithm (FIND-AF) on more than 1.5 million individuals registered in UK primary care to predict the risk of incident AF within the next 6 months (figure 4). When tested in over 400 000 individuals, FIND-AF demonstrated good predictive accuracy, which was superior to other risk scores and robust in both sexes and across ethnic groups. FIND-AF identified a cohort of younger people at higher risk of AF and more efficiently identified individuals diagnosed with AF within 6 months compared with age-based risk stratification. Finally, short-term predicted AF risk also translated to long-term AF occurrence.

Current approaches to targeting investigation for undiagnosed AF are based on age.^7^ Our analysis demonstrated that one-fifth of newly detected AF cases within 6 months occur in people aged ≤65 years, emphasising the opportunity lost when enhanced AF investigation is restricted to older populations. ECGs can be used to accurately predict AF risk,^20^ but they are not widely available in the community, whereas 98% of the UK population are registered in primary care with an accompanying EHR.^8^ Our meta-analysis of AF prediction algorithms using EHRs demonstrated that algorithms developed using traditional regression techniques provided only moderate discrimination performance.^10^ In our study, a machine learning prediction algorithm (FIND-AF) outperformed the C_2_HEST and CHA_2_DS_2_-VASc scores.

For a machine learning prediction algorithm to be useful in clinical practice, it must be implementable within the clinical workflow, provide prediction that meaningfully informs decision-making and engender confidence in how outputs were arrived at.^21^ FIND-AF has been designed to be implemented and displayed through EHR systems, so will be available in a platform that healthcare professionals are interacting with as part of routine care. By design, FIND-AF provides AF risk prediction over a short time frame and so could assist clinicians at point of care in identifying patients for targeted diagnostics such as ECG monitoring. Finally, the most important predictors in FIND-AF are already well-recognised risk factors for AF (for example, age, heart failure, valvular heart disease), which provide reassurance in the associations being made by the algorithm.^7^

Fairness is a critical characteristic when considering the impact of prediction algorithms in healthcare. The CHARGE-AF and PuLSE-AI algorithms have strong AF prediction performance,^9 11^ yet incorporate variables that are frequently missing (height, weight and systolic and diastolic blood pressure).^10^ Consequently, their applicability is limited to 17% and 35% of primary care EHRs, respectively.^9 11^ Often, health data poverty disproportionately affects individuals from minority ethnicities and deprived backgrounds, so the application of these algorithms could reinforce health inequities.^22^ Furthermore, whether their performance varies by sex and in minority ethnic groups in European populations is unknown. In our study, the C_2_HEST and CHA_2_DS_2_-VASc scores were less accurate in women compared with men, and their performance varied substantially across different ethnic groups. FIND-AF’s design enabled its application to every single patient record in a nationally representative dataset of routinely collected primary care EHRs; and performance was robust in both sexes and across minority ethnic groups.

Three barriers need to be overcome for FIND-AF to be accepted into clinical practice. First, it requires external validation, which is currently underway using The Phoenix Partnership UK primary care EHR system (ResearchOne) and the Israeli Clalit Health Services. Second, prospective validation of FIND-AF is critical before implementation into clinical practice. We are launching a pilot implementation study across primary care sites where individuals identified at higher risk will be offered rhythm monitoring (The BHF Bristol Myers Squibb Cardiovascular Catalyst Award—CC/22/250026). Third, a cost utility analysis and budget impact analysis of the use of FIND-AF will need to be conducted.

Primary care EHRs in the UK are nationwide and held centrally, so FIND-AF could be activated at scale across geographically disparate sites to identify a subpopulation at elevated AF risk. The cohort identified as higher risk in this study included younger people who would currently be excluded from screening pathways, and higher predicted AF risk was associated with elevated AF occurrence both in the short and long term. Therefore, FIND-AF could facilitate efficient population-based AF screening or comprehensive programmes designed to improve risk factor profiles (including targeted weight loss and optimisation of blood pressure control).^23^

Screening for AF would adhere to many of the Wilson and Junger principles for a screening programme.^24^ Opportunistic screening guided by age has not been demonstrated to increase AF detection rates,^25^ but this may change in a more precisely defined higher-risk cohort. Systematic screening of older patients with intermittent or continuous (invasive or non-invasive) rhythm monitors is associated with increased AF detection rates, compared with routine care.^24^ However, the yield of new cases is low (3% in the STROKESTOP trial)^26^ and in our study, FIND-AF more efficiently identified a cohort with a higher rate of clinically detected AF than age-based approaches. Accurate risk assessment would be an integral component of a systematic screening process but ongoing research is needed to address the issues of the effectiveness and safety of treatment of screen-detected AF, and the costs of widespread use of ECG monitoring and prescription of oral anticoagulation, after the mixed results of the recently published LOOP and STROKESTOP trials.^26 27^

There are some limitations to our study. First, the CPRD database is routinely collected, retrospective primary care data. Underestimation of AF incidence is possible since there will have been individuals with unrecorded asymptomatic AF. Second, important predictor variables may have been ‘missing by design’; nonetheless, we aimed to develop an algorithm that used routinely recorded data. Third, our choice of an RF classifier was based on a systematic review of AF prediction in EHRs,^10^ and it is possible other machine learning methods may have performed differently in our study. Fourth, the algorithm will need to be updated as population characteristics change, data quality of EHRs improves and new or additional risk factors emerge. Fifth, electrophysiology procedures not specified as treating AF (including pacemaker implantations and percutaneous ablations) were a strong predictor of AF risk, and this may be a result of detection bias.

## Conclusions

We trained and tested a novel machine learning algorithm (FIND-AF) that was applicable at scale within a nationwide routinely collected primary care EHR dataset. FIND-AF was able to accurately predict AF risk within 6 months and identify a cohort at elevated risk of AF in the longer term.

## Data Availability

Data may be obtained from a third party and are not publicly available. Data used in this study can be accessed through CPRD subject to protocol approval. The algorithm can be shared with researchers who agree to use it only for research purposes with a data sharing agreement.

## References

[1] Wu J , Nadarajah R , Nakao YM , et al . Temporal trends and patterns in atrial fibrillation incidence: a population-based study of 3·4 million individuals. Lancet Reg Health Eur 2022;17:100386. 10.1016/j.lanepe.2022.100386 35721699 PMC9198843

[2] Svennberg E , Engdahl J , Al-Khalili F , et al . Mass screening for untreated atrial fibrillation: the STROKESTOP study. Circulation 2015;131:2176–84. 10.1161/CIRCULATIONAHA.114.014343 25910800

[3] Kamel H . Cryptogenic stroke and atrial fibrillation. N Engl J Med 2014;371:1261–2. 10.1056/NEJMc1409495 25259388

[4] Ruff CT , Giugliano RP , Braunwald E , et al . Comparison of the efficacy and safety of new oral anticoagulants with warfarin in patients with atrial fibrillation: a meta-analysis of randomised trials. Lancet 2014;383:955–62. 10.1016/S0140-6736(13)62343-0 24315724

[5] Kirchhof P , Camm AJ , Goette A , et al . Early rhythm-control therapy in patients with atrial fibrillation. N Engl J Med 2020;383:1305–16. 10.1056/NEJMoa2019422 32865375

[6] NHS . Cardiovascular disease. 2019. Available: https://www.longtermplan.nhs.uk/areas-of-work/cardiovascular-disease/

[7] Hindricks G , Potpara T , Dagres N , et al . 2020 ESC guidelines for the diagnosis and management of atrial fibrillation developed in collaboration with the european association for cardio-thoracic surgery (EACTS): the task force for the diagnosis and management of atrial fibrillation of the european society of cardiology (ESC) developed with the special contribution of the european heart rhythm association (EHRA) of the ESC. Eur Heart J 2021;42:373–498. 10.1093/eurheartj/ehaa612 32860505

[8] Herrett E , Gallagher AM , Bhaskaran K , et al . Data resource profile: clinical practice research Datalink (CPRD). Int J Epidemiol 2015;44:827–36. 10.1093/ije/dyv098 26050254 PMC4521131

[9] Himmelreich JCL , Lucassen WAM , Harskamp RE , et al . CHARGE-AF in a national routine primary care electronic health records database in the Netherlands: validation for 5-year risk of atrial fibrillation and implications for patient selection in atrial fibrillation screening. Open Heart 2021;8:e001459. 10.1136/openhrt-2020-001459 33462107 PMC7816907

[10] Nadarajah R , Alsaeed E , Hurdus B , et al . Prediction of incident atrial fibrillation in community-based electronic health records: a systematic review with meta-analysis. Heart 2022;108:1020–9. 10.1136/heartjnl-2021-320036 34607811 PMC9209680

[11] Hill NR , Ayoubkhani D , McEwan P , et al . Predicting atrial fibrillation in primary care using machine learning. PLoS One 2019;14:e0224582. 10.1371/journal.pone.0224582 31675367 PMC6824570

[12] Ruigómez A , Johansson S , Wallander MA , et al . Incidence of chronic atrial fibrillation in general practice and its treatment pattern. J Clin Epidemiol 2002;55:358–63. 10.1016/s0895-4356(01)00478-4 11927203

[13] Collins GS , Reitsma JB , Altman DG , et al . Transparent reporting of a multivariable prediction model for individual prognosis or diagnosis (TRIPOD): the TRIPOD statement. The TRIPOD group. Circulation 2015;131:211–9. 10.1161/CIRCULATIONAHA.114.014508 25561516 PMC4297220

[14] Kotecha D , Asselbergs FW , Achenbach S , et al . CODE-EHR best practice framework for the use of structured electronic healthcare records in clinical research. BMJ 2022;378:e069048. 10.1136/bmj-2021-069048 36562446 PMC9403753

[15] Breiman L . Random forests. Mach Learn 2001;45:5–32. 10.1023/A:1010933404324

[16] Routen A , Akbari A , Banerjee A , et al . Strategies to record and use ethnicity information in routine health data. Nat Med 2022;28:1338–42. 10.1038/s41591-022-01842-y 35641824

[17] Groenwold RHH . Informative missingness in electronic health record systems: the curse of knowing. Diagn Progn Res 2020;4:8.:8. 10.1186/s41512-020-00077-0 32699824 PMC7371469

[18] Sakamoto Y , Ishiguro M , Kitagawa G . Akaike information criterion statistics. Dordrecht Netherlands D Reidel 1986;81:26853.

[19] Szymanski T , Ashton R , Sekelj S , et al . Budget impact analysis of a machine learning algorithm to predict high risk of atrial fibrillation among primary care patients. Europace 2022;24:1240–7. 10.1093/europace/euac016 35226101

[20] Attia ZI , Noseworthy PA , Lopez-Jimenez F , et al . An artificial intelligence-enabled ECG algorithm for the identification of patients with atrial fibrillation during sinus rhythm: a retrospective analysis of outcome prediction. Lancet 2019;394:861–7. 10.1016/S0140-6736(19)31721-0 31378392

[21] van Smeden M , Heinze G , Van Calster B , et al . Critical appraisal of artificial intelligence-based prediction models for cardiovascular disease. Eur Heart J 2022;43:2921–30. 10.1093/eurheartj/ehac238 35639667 PMC9443991

[22] Ibrahim H , Liu X , Zariffa N , et al . Health data poverty: an assailable barrier to equitable digital health care. Lancet Digit Health 2021;3:e260–5. 10.1016/S2589-7500(20)30317-4 33678589

[23] Middeldorp ME , Pathak RK , Meredith M , et al . Prevention and regressive effect of weight-loss and risk factor modification on atrial fibrillation: the REVERSE-AF study. Europace 2018;20:1929–35. 10.1093/europace/euy117 29912366

[24] Jones NR , Taylor CJ , Hobbs FDR , et al . Screening for atrial fibrillation: a call for evidence. Eur Heart J 2020;41:1075–85. 10.1093/eurheartj/ehz834 31811716 PMC7060457

[25] Uittenbogaart SB , Verbiest-van Gurp N , Lucassen WAM , et al . Opportunistic screening versus usual care for detection of atrial fibrillation in primary care: cluster randomised controlled trial. BMJ 2020;370:m3208. 10.1136/bmj.m3208 32938633 PMC7492823

[26] Svennberg E , Friberg L , Frykman V , et al . Clinical outcomes in systematic screening for atrial fibrillation (STROKESTOP): a multicentre, parallel group, unmasked, randomised controlled trial. Lancet 2021;398:1498–506. 10.1016/S0140-6736(21)01637-8 34469764

[27] Svendsen JH , Diederichsen SZ , Højberg S , et al . Implantable loop recorder detection of atrial fibrillation to prevent stroke (the loop study): a randomised controlled trial. Lancet 2021;398:1507–16. 10.1016/S0140-6736(21)01698-6 34469766

