# Supplementary material for: Prediction of short-term atrial fibrillation risk using primary care electronic health records
Source: Heart. 2023 Feb 9;109(14):1072–9. doi: 10.1136/heartjnl-2022-322076 (PMC10359547; doi:10.1136/heartjnl-2022-322076)
Supplement: Supplementary data [file heartjnl-2022-322076supp001.pdf]

Supplementary Appendix

Prediction of short-term atrial fibrillation risk using primary care electronic health records

Ramesh Nadarajah*, Jianhua Wu*, David Hogg, Keerthenan Raveendra, Yoko M Nakao, Kazuhiro Nakao, John Parry, Chris Bates, Ronen Arbel, Moti Haim, Doron Zahger, Campbell Cowan, Chris P Gale

Supplementary Introduction 2

Supplementary Table S1. Algorithms that have been derived and/or validated in community-based EHR for predicting AF 2

Supplementary Table S2. Algorithms that have been derived and/or validated in European community-based EHRs for predicting AF 4

Supplementary Methods 5

Supplementary Table S3. Read codes and ICD-10 codes used to define the outcomes of atrial fibrillation or atrial flutter 5

Training of the Random Forest classifier 6

Supplementary Table S4. Baseline demographic and comorbidity variables used in algorithms tested for predicting incident AF in community-based electronic health records 7

Supplementary Table S5. Candidate variables added after literature search with accompanying reference demonstrating association 9

Supplementary Table S6. Variable categorisations with rationale 12

Supplementary Results 16

Supplement Table S7. Baseline characteristics of training and testing datasets 16

Supplementary Table S8. Net reclassification using FIND-AF 17

Supplement Figure S1. Calibration plots 20

Supplementary Figure S2. Decision curve analysis for FIND-AF versus CHA_2DS_2-VASc and C_2HEST 22

References 23

Nadarajah R, *et al. Heart* 2023; 109:1072–1079. doi: 10.1136/heartjnl-2022-322076

Supplementary Introduction

Supplementary Table S1. Algorithms that have been derived and/or validated in community-based EHR for predicting AF

Algorithm	Study Aim	Study	EHR cohort (country)	Age eligibility (years)	Discrimination (c-statistic)	Follow-up	Variable frequently missing in routinely-collected primary care EHR
**Models originally derived for another purpose but tested for prediction of incident atrial fibrillation**							
$CHADS_2$	EV	Chao 2013	NHIRD (TW)	≥18	0.713	10	N/A
	EV	Saliba 2016	ClalitHS (IL)	≥50	0.728	3	
	EV	Li 2019	YMID (CN)	≥18	0.632	11	
	EV	Li 2019	NHIS-HEALS (KR)	≥18	0.637	11	
	EV	Kim 2020	NHIS-NSC (KR)	≥18	0.652	5	
CHA_2DS_2-VASc	EV	Saliba 2016	ClalitHS (IL)	≥50	0.744	3	N/A
	EV	Li 2019	YMID (CN)	≥18	0.687	11	
	EV	Li 2019	NHIS-HEALS (KR)	≥18	0.637	11	
	EV	Himmelreich 2020	Nivel-PCD (NL)	≥40	0.669	5	
	EV	Kim 2020	NHIS-NSC (KR)	≥18	0.654	5	
HATCH	EV	Suenari 2017	NHIRD (TW)	≥20	0.716	9	N/A
	EV	Li 2019	YMID (CN)	≥18	0.633	11	
	EV	Li 2019	NHIS-HEALS (KR)	≥18	0.646	11	
	EV	Kim 2020	NHIS-NSC (KR)	≥18	0.669	5	
	EV	Hu-WS 2020	NHIRD (TW)	≥18	0.771	14	
**Machine Learning models**							
Pfizer-AI	D	Hill 2019	CPRD (UK)	≥30	0.827	11	Height, weight, BMI, SBP, DBP
	EV	Sekelj 2020	Discover (UK)	≥30	0.870	8	
NHIRD	D	Hu-WS 2019	NHIRD (TW)	≥18	0.948	14	Follow-up duration (years)
NHIS-NSC	D	Kim 2020	NHIS-NSC (KR)	≥18	0.845	5	BMI, SBP, Triglycerides, total cholesterol, HDL cholesterol, LDL cholesterol, eGFR, GGT, fasting blood glucose, Haemoglobin, AST, Socioeconomic status
**Regression Models derived in electronic health records**							

Nadarajah R, *et al. Heart* 2023; 109:1072–1079. doi: 10.1136/heartjnl-2022-322076

C_2HEST	D	Li 2019	YMID (CN)	≥18	0.750	11	N/A
	EV	Li 2019	NHIS-HEALS (KR)	≥18	0.654	11	
	EV	Hu-WS 2020	NHIRD (TW)	≥18	0.790	14	
	EV	Lip 2020	DCRS, DNPR, DPR (DK)	65	0.588	5	
				70	0.594		
				75	0.593		
MHS	D	Aronson 2018	MHS (IL)	≥50	0.743	10	BMI, SBP
Taiwan AF	D	Chao 2021	NHIRD (TW)	≥40	0.857	1	N/A
					0.825	5	
					0.797	10	
					0.756	16	
InGef	D	Schnabel 2022	InGef (G)	≥45	0.829	1	N/A
**Regression model derived in a prospective cohort design**							
CHARGE-AF	EV	Hill 2019	CPRD (UK)	≥30	0.725	11	Height, weight, SBP, DBP

Nadarajah R, *et al. Heart* 2023; 109:1072–1079. doi: 10.1136/heartjnl-2022-322076

BMJ Publishing Group Limited (BMJ) disclaims all liability and responsibility arising from any reliance placed on this supplemental material which has been supplied by the author(s)

Supplementary Table S2. Algorithms that have been derived and/or validated in European community-based EHRs for predicting AF

Algorithm	Study Aim	Study	EHR cohort (country)	Age eligibility (years)	Discrimination (c-statistic)	Follow-up	Variable frequently missing in routinely-collected primary care EHR
**Models originally derived for another purpose but tested for prediction of incident atrial fibrillation**							
CHA_2DS_2-VASc	EV	Himmelreich 2020	Nivel-PCD (NL)	≥40	0.669	5	N/A
**Machine Learning models**							
CPRD	D	Hill 2019	CPRD (UK)	≥30	0.827	11	Height, weight, BMI, SBP, DBP
	EV	Sekelj 2020	Discover (UK)	≥30	0.870	8	
**Regression Models derived in electronic health records**							
C_2HEST	EV	Lip 2020	DCRS, DNPR, DPR (DK)	65	0.588	5	N/A
				70	0.594		
				75	0.593		
InGef	D	Schnabel 2022	InGef (G)	≥45	0.829	1	N/A
**Regression model derived in a prospective cohort design**							
CHARGE-AF	EV	Hill 2019	CPRD (UK)	≥30	0.725	11	Height, weight, SBP, DBP

AF, Atrial Fibrillation; $CHADS_2$, Congestive heart failure, Hypertension, Age >75, Diabetes mellitus, prior Stroke or transient ischemic attack [2 points]; CHA_2DS_2-VASc, Congestive heart failure, Hypertension, Age >75 [2 points], Stroke/transient ischemic attack/thromboembolism [2 points], Vascular disease, Age 65-74, Sex Category; CHARGE-AF, Cohorts for Heart and Aging Research in Genomic Epidemiology; C_2HEST, Coronary artery disease / Chronic obstructive pulmonary disease [1 point each], Hypertension, Elderly (Age ≥75, 2 points), Systolic heart failure, Thyroid disease (hyperthyroidism); ClalitHS, Clalit Health Services; CPRD, Clinical Practice Research Datalink; D, derivation; DCRS, Danish Civil Registration system; DK, Denmark; DNPR, Danish National Patient Register; DPR, Danish Prescription Regster; EHR, electronic health record; EV, external validation; G, Germany; HATCH, Hypertension, Age, stroke or Transient ischemic attack, Chronic obstructive pulmonary disease, Heart failure; IL, Israel; KR, Republic of Korea; MHS, Maccabi Healthcare Services; NHIRD, National Health Insurance Research Database; NHIS-HEALS, National Health Insurance Service - Health screening Cohort; NHIS-NSC, National Health Insurance Service-based National Sample Cohort; Nivel-PCD, Netherlands Institute for Health Services Research Primary Care Database; NL, Netherlands; TW, Taiwan; UK, United Kingdom; YMID, Yunnan Medical Insurance Database.

Nadarajah R, *et al. Heart* 2023; 109:1072–1079. doi: 10.1136/heartjnl-2022-322076

Supplementary Methods

Supplementary Table S3. Read codes and ICD-10 codes used to define the outcomes of atrial fibrillation or atrial flutter

**Code**	**Description**
Readcodes	
G573200	Paroxysmal atrial fibrillation
G573400	Permanent atrial fibrillation
G573500	Persistent atrial fibrillation
3272	ECG: atrial fibrillation
G573000	Atrial fibrillation
G573300	Non-rheumatic atrial fibrillation
G573.00	Atrial fibrillation and flutter
G573z00	Atrial fibrillation and flutter NOS
3273	ECG: atrial flutter
G573100	Atrial flutter
ICD-10 codes	
I48	Atrial fibrillation and flutter

Training of the Random Forest classifier

Each decision tree used Gini impurity, commonly used in classification and regression tree (CART) algorithms, to measure the split quality.[1] The minimum impurity split threshold for each node, above which a node will split into two or more branches, was set to 10^{-7}. The minimum number of samples required to split a node was set to two. The minimum samples per leaf was set to one. All the algorithm's hyperparameters were tuned using the grid search method, in which all possible combinations were evaluated, resulting in 1000 trees, mtry = 8 (the number of random features to consider in each tree) and nodesize = 12 (number of patients classified at that node).

Nadarajah R, *et al. Heart* 2023; 109:1072–1079. doi: 10.1136/heartjnl-2022-322076

Supplementary Table S4. Baseline demographic and comorbidity variables used in algorithms tested for predicting incident AF in community-based electronic health records

Algorithm	Demographics	Comorbidities
$CHADS_2$	Age	Hypertension, CHF, diabetes mellitus, CVA
CHA_2DS_2-VASc	Age, sex	Hypertension, CHF, stroke/TIA/thromboembolism, vascular disease
CHARGE-AF	Age, race, smoking status	Anti-hypertensive medication, MI, CHF, DM
C_2HEST	Age	Hypertension, ischaemic heart disease, CHF, COPD, thyroid disease
HATCH	Age	Hypertension, CHF, stroke/TIA, COPD
InGef	Age, sex	Anti-hypertension medication, heart failure medication, chronic kidney disease, disorderd of lipoprotein metabolism and other lipidaemias, pulmonary heart diseases cardiac arrhythmias, other cerebrovascular disease, diverticular disease of intestine, dorsalgia, breathing abnormalities
MHS	Age, sex	Anti-hypertensive medication, MI, CHF, peripheral vascular disease, inflammatory disease in a female, COPD
NHIRD	Age (years), age group, sex	Hypertension, CHF, COPD, rheumatological disease, dyslipidaemia, DM, CVA or TIA, sleep disorder, cancer, hyperthyroidism, vascular disease, gout, CKD or ESRD, anaemia
NHIS-NSC*	Age, sex, smoking (pack-year), alcohol	Hypertension, CHF, MI, vascular disease, stroke/TIA, COPD
Pfizer-AI	Age, sex, race, smoking status	Hypertension, anti-hypertensive medication, CHF, congenital heart disease, MI, LVH, type 1 DM, type 2 DM
Taiwan AF	Age, sex, alcohol excess	Hypertension, CHF, IHD, ESRD

AF, Atrial Fibrillation; $CHADS_2$, Congestive heart failure, Hypertension, Age >75, Diabetes mellitus, prior Stroke or transient ischemic attack [2 points]; CHA_2DS_2-VASc, Congestive heart failure, Hypertension, Age >75 [2 points], Stroke/transient ischemic attack/thromboembolism [2 points]; CHARGE-AF, Cohorts for Heart and Aging Research in Genomic Epidemiology; C_2HEST, Coronary artery disease / Chronic obstructive pulmonary disease [1 point each], Hypertension, Elderly (Age ≥75, 2 points), Systolic heart failure, Thyroid disease (hyperthyroidism); CHF, chronic heart failure; CKD, chronic kidney disease; COPD, chronic obstructive pulmonary disease; CPRD, Clinical Practice Research Datalink; CVA, cerebrovascular accident; DM, diabetes mellitus; ESRD, end-stage renal disease; HATCH, Hypertension, Age, stroke or Transient ischemic attack, Chronic obstructive pulmonary disease, Heart failure; IHD, ischaemic heart disease; LVH, left ventricular hypertrophy; MHS, Maccabi Healthcare Services; MI, myocardial infarction; NHIRD, National Health Insurance Research Database; NHIS-HEALS, National Health Insurance Service - Health screening Cohort; NHIS-NSC, National Health Insurance Service-based National Sample Cohort; TIA, transient ischaemic attack.

Nadarajah R, *et al. Heart* 2023; 109:1072–1079. doi: 10.1136/heartjnl-2022-322076

* In Kim 2020 prediction model development using machine learning was completed both with and without the predictor $PM_{2.5}$ - which is fine particular matter air pollution. In this analysis we have only included the model without $PM_{2.5}$ as it is judged not to be a predictor that would be routinely available in primary care or population EHR.

Supplementary Table S5. Candidate variables added after literature search with accompanying reference demonstrating association

**Comorbidity associated with / predictive of atrial fibrillation**	**Categorisation**	**Reference demonstrating association with AF and rationale for categorisation**
Cardiac surgery	Valvular,	Greenberg JW, Lancaster TS, Schuessler RB, et al. Postoperative atrial fibrillation following cardiac surgery: a persistent complication. Eur J Cardiothorac Surg 2017;52(4):665-72.<br><br>Within overall cardiac surgical procedures incidence of post-operative AF is 35%, isolated CABG has an incidence of 20—30% and isolated valve surgeries have an incidence of 35-40
	Non-valvular	
Deep venous thrombosis	-	Lutsey P, Norby F, Alonso A, et al. Atrial fibrillation and venous thromboembolism: evidence of bidirectionality in the Atherosclerosis Risk in Communities Study. J Thromb Haemost 2018;16(4):670-79.
Infective Endocarditis	-	Ferrera C, Vilacosta I, Fernández C, et al. Usefulness of new-onset atrial fibrillation, as a strong predictor of heart failure and death in patients with native left-sided infective endocarditis. The American journal of cardiology 2016;117(3):427-33.
Electrophysiology procedure affecting the atria	-	Strickberger SA, Man KC, Daoud EG, et al. Adenosine-induced atrial arrhythmia: a prospective analysis. Ann Intern Med 1997;127(6):417-22.<br><br>Khachab, H., and B. Brembilla-Perrot. "Prevalence of atrial fibrillation in patients with history of paroxysmal supraventricular tachycardia." International journal of cardiology 166.1 (2013): 221-224.
Hypertrophic cardiomyopathy	-	Siontis KC, Geske JB, Ong K, et al. Atrial fibrillation in hypertrophic cardiomyopathy: prevalence, clinical correlations, and mortality in a large high-risk population. Journal of the American Heart Association 2014;3(3):e001002.
Inflammatory bowel disease	-	Boos CJ. Infection and atrial fibrillation: inflammation begets AF. Eur Heart J 2020
Intensive care unit admission	-	Klein Klouwenberg PM, Frencken JF, Kuipers S, et al. Incidence, predictors, and outcomes of new-onset atrial fibrillation in critically ill patients with sepsis. A cohort study. Am J Respir Crit Care Med 2017;195(2):205-11.
Infection	Gastrointestinal	Gundlund A, Olesen JB, Butt JH, et al. One-year outcomes in atrial fibrillation presenting during infections: a nationwide registry-based study. Eur Heart J 2020;41(10):1112-19.<br><br>Chang T-Y, Chao T-F, Liu C-J, et al. The association between influenza infection, vaccination, and atrial fibrillation: A nationwide case-control study. Heart Rhythm 2016;13(6):1189-94.<br><br>Klein Klouwenberg PM, Frencken JF, Kuipers S, et al. Incidence, predictors, and outcomes of new-onset atrial fibrillation in critically ill patients with sepsis. A cohort study. Am J Respir Crit Care Med
	Influenza	
	Respiratory	
	Sepsis	

		2017;195(2):205-11.<br><br>In a cohort study among infections precipitating AF the order of risk is as follows: Pneumonia > sepsis > urinary tract infection > gastrointestinal infection
	Urinary	
Myocarditis	-	Wang Z, Wang Y, Lin H, et al. Early characteristics of fulminant myocarditis vs non-fulminant myocarditis: a meta-analysis. Medicine 2019;98(8)
Pulmonary embolus	-	Ptaszynska-Kopczynska K, Kiluk I, Sobkowicz B. Atrial fibrillation in patients with acute pulmonary embolism: clinical significance and impact on prognosis. BioMed research international 2019;2019
Pericarditis	-	Imazio M, Lazaros G, Picardi E, et al. Incidence and prognostic significance of new onset atrial fibrillation/flutter in acute pericarditis. Heart 2015;101(18):1463-67.
Pulmonary hypertension	-	Olsson KM, Nickel NP, Tongers J, et al. Atrial flutter and fibrillation in patients with pulmonary hypertension. Int J Cardiol 2013;167(5):2300-05.
Surgery (non-cardiac)	Colorectal	Siu CW, Tung HM, Chu KW, et al. Prevalence and predictors of new-onset atrial fibrillation after elective surgery for colorectal cancer. Pacing Clin Electrophysiol 2005;28:S120-S23.<br><br>Onaitis M, D'Amico T, Zhao Y, et al. Risk factors for atrial fibrillation after lung cancer surgery: analysis of the Society of Thoracic Surgeons general thoracic surgery database. The Annals of thoracic surgery 2010;90(2):368-74.<br><br>Philip I, Berroëta C, Leblanc I. Perioperative challenges of atrial fibrillation. Current Opinion in Anesthesiology 2014;27(3):344-52.<br><br>Thoracic surgery is associated with the greatest risk of post-operative AF amongst non-cardiac surgeries followed by colorectal then vascular surgery
	Thoracic	
	Vascular	
Valvular heart disease	Mitral stenosis / rheumatic valvular disease	Iung B, Leenhardt A, Extramiana F. Management of atrial fibrillation in patients with rheumatic mitral stenosis. Heart 2018;104(13):1062-68.<br><br>Levy S. Factors predisposing to the development of atrial fibrillation. Pacing Clin Electrophysiol 1997;20(10):2670-74.<br><br>Grigioni F, Avierinos J-F, Ling LH, et al. Atrial fibrillation complicating the course of degenerative mitral regurgitation: determinants and long-term outcome. J Am Coll Cardiol 2002;40(1):84-92.<br><br>The association of mitral stenosis and rheumatic valve disease with AF is greater than mitral regurgitation followed by diseases of other valves
	Non-mitral valve / other valves	
	Mitral regurgitation	
Vascular dementia	-	Ott A, Breteler MM, De Bruyne MC, et al. Atrial fibrillation and dementia in a population-based study: the Rotterdam Study. Stroke 1997;28(2):316-21.
Weight	Obese	Lavie CJ, Pandey A, Lau DH, et al. Obesity and atrial fibrillation prevalence, pathogenesis, and prognosis:
	Overweight	

	Under-weight	effects of weight loss and exercise. J Am Coll Cardiol 2017;70(16):2022-35.<br><br>Frost L, Hune LJ, Vestergaard P. Overweight and obesity as risk factors for atrial fibrillation or flutter: the Danish Diet, Cancer, and Health Study. The American journal of medicine 2005;118(5):489-95.<br><br>Lee S-R, Choi E-K, Park CS, et al. Direct oral anticoagulants in patients with nonvalvular atrial fibrillation and low body weight. J Am Coll Cardiol 2019;73(8):919-31.<br><br>Obesity is associated with a greater risk of AF than being overweight. Low body weight is associated with a higher risk of AF than normal weight.

Supplementary Table S6. Variable categorisations with rationale

**Comorbidity associated with / predictive of atrial fibrillation**	**Categorisation**	**References and Rationale for categorisation**
Demographics		
Age	-	Hindricks G, Potpara T, Dagres N, et al. 2020 ESC Guidelines for the diagnosis and management of atrial fibrillation developed in collaboration with the European Association of Cardio-Thoracic Surgery (EACTS). Eur Heart J 2020<br><br>Incidence of AF increases with age (therefore included as a continuous variable)
Sex	Men	Hindricks G, Potpara T, Dagres N, et al. 2020 ESC Guidelines for the diagnosis and management of atrial fibrillation developed in collaboration with the European Association of Cardio-Thoracic Surgery (EACTS). Eur Heart J 2020<br><br>AF is more common in men
	Women	
Ethnicity	Asian	Shen AY-J, Contreras R, Sobnosky S, et al. Racial/ethnic differences in the prevalence of atrial fibrillation among older adults—a cross-sectional study. J Natl Med Assoc 2010;102(10):906-14.<br><br>Chiang C-E, Zhang S, Tse HF, et al. Atrial fibrillation management in Asia: from the Asian expert forum on atrial fibrillation. Int J Cardiol 2013;164(1):21-32.<br><br>White, Asian, pacific Asian, and black ethnicities have different odds ratios of development of AF
	Black	
	Mixed	
	Other	
	Pacific Asian	
	White	
Alcohol use	Ex-	Samokhvalov AV, Irving HM, Rehm J. Alcohol consumption as a risk factor for atrial fibrillation: a systematic review and meta-analysis. European Journal of Preventive Cardiology 2010;17(6):706-12.<br><br>There is a monotonic dose-response relationship between alcohol consumption and AF incidence
	Light,	
	Moderate	
	Excess	
	Unspecified	
Smoking	Current	Heeringa J, Kors JA, Hofman A, et al. Cigarette smoking and risk of atrial fibrillation: the Rotterdam Study. Am Heart J 2008;156(6):1163-69.<br><br>Watanabe I. Smoking and risk of atrial fibrillation: Elsevier, 2018.<br><br>Current and ex-smokers are at increased risk of AF, with a higher risk in current smokers.
	Ex	
Weight	Obese	See table S4
	Overweight	
	Under-weight	
Comorbidities		
Adult congenital heart disease	-	-
Anaemia	-	-
Cancer	Leukaemia	Thompson PA, Lévy V, Tam CS, et al. Atrial fibrillation in CLL patients treated with ibrutinib. An international retrospective study. Br J Haematol 2016;175(3):462-66.
	Lymphoma	
	Metastasis	

	Skin cancers other than melanoma	Sorigue M, Gual-Capllonch F, Garcia O, et al. Incidence, predictive factors, management, and survival impact of atrial fibrillation in non-Hodgkin lymphoma. Ann Hematol 2018;97(9):1633-40.<br><br>Han H, Chen L, Lin Z, et al. Prevalence, trends, and outcomes of atrial fibrillation in hospitalized patients with metastatic cancer: findings from a national sample. Cancer medicine 2021;10(16):5661-70.<br><br>AF risk is higher in patients with leukaemia and lymphoma, especially treated with iritunib. Solid organ cancers (such as lung and colorectal cancer) are more likely to undergo surgery. Metastatic disease is associated with higher risk of AF compared to non-metastatic disease. Skin cancers other than melanoma have a lower risk of metastasis and hence AF.
	Solid organ	
Cardiac surgery	Valvular,	See table S4
	Non-valvular	
Chronic kidney disease	Stage 1-2	Alonso A, Lopez FL, Matsushita K, et al. Chronic kidney disease is associated with the incidence of atrial fibrillation: the Atherosclerosis Risk in Communities (ARIC) study. Circulation 2011;123(25):2946-53.<br><br>Risk of AF increases as CKD stage worsens and if there is proteinuria
	Stage 3	
	Stage 4	
	Stage 5	
	Unspecified	
	Other	
COPD	-	-
Cerebro-vascular accident	Intracerebral haemorrhage	Hindricks G, Potpara T, Dagres N, et al. 2020 ESC Guidelines for the diagnosis and management of atrial fibrillation developed in collaboration with the European Association of Cardio-Thoracic Surgery (EACTS). Eur Heart J 2020<br><br>Association with AF is higher for ischaemic strokes than haemorrhagic strokes
	Subarachnoid haemorrhage	
	Unspecified	
Diabetes Mellitus	Good control	Dublin S, Glazer NL, Smith NL, et al. Diabetes mellitus, glycemic control, and risk of atrial fibrillation. J Gen Intern Med 2010;25(8):853-58.<br><br>Poorer glycaemic control is associated with a higher risk of AF compared to better glycaemic control or no diabetes
	Poor control	
	Unspecified / secondary	
Deep venous thrombosis	-	-
Dyslipidaemia	-	-
Infective Endocarditis	-	-
Electrophysiology procedure affecting the atria	-	-
Gout	-	-
Hypertrophic cardiomyopathy	-	-
Heart failure	-	-
Hypertension	Poor control	Dzeshka MS, Shantsila A, Shantsila E, et al. Atrial fibrillation and hypertension. Hypertension 2017;70(5):854-61.<br><br>Poorer control of hypertension and end organ damage is
	Unspecified / secondary	

Nadarajah R, *et al. Heart* 2023; 109:1072–1079. doi: 10.1136/heartjnl-2022-322076

		associated with a higher risk of developing AF
Hyperthyroidism	-	-
Inflammatory bowel disease	-	-
Intensive care unit admission	-	-
Ischaemic heart disease	Chronic	Huxley RR, Lopez FL, Folsom AR, et al. Absolute and attributable risks of atrial fibrillation in relation to optimal and borderline risk factors: the Atherosclerosis Risk in Communities (ARIC) study. Circulation 2011;123(14):1501-08.<br><br>Pizzetti F, Turazza F, Franzosi M, et al. Incidence and prognostic significance of atrial fibrillation in acute myocardial infarction: the GISSI-3 data. Heart 2001;86(5):527-32.<br><br>There is a high risk of AF in the acute setting of myocardial infarction as well as evidence in the context of underlying chronic coronary syndromes.
	Myocardial infarction	
	Percutaneous coronary intervention	
Infection	Gastrointestinal	See table S4
	Influenza	
	Respiratory	
	Sepsis	
	Urinary	
Left ventricular hypertrophy	-	-
Myocarditis	-	-
Obstructive sleep apnoea	-	-
Pulmonary embolus	-	-
Pericarditis	-	-
Pulmonary hypertension	-	-
Peripheral vascular disease	-	-
Rheumatological condition	Autoimmune connective tissue diseases	Lee E, Choi E-K, Jung J-H, et al. Increased risk of atrial fibrillation in patients with Behçet's disease: a nationwide population-based study. Int J Cardiol 2019;292:106-11.<br><br>Moon I, Choi E-K, Jung J-H, et al. Ankylosing spondylitis: a novel risk factor for atrial fibrillation—a nationwide population-based study. Int J Cardiol 2019;275:77-82.<br><br>Melduni RM, Cooper LT, Gersh BJ, et al. Association of Autoimmune Vasculitis and Incident Atrial Fibrillation: A Population-Based Case-Control Study. Journal of the American Heart Association 2020;9(18):e015977.<br><br>Naaraayan A, Meredith A, Nimkar A, et al. Arrhythmia prevalence among patients with Polymyositis-Dermatomyositis in the United States: an observational study. Heart Rhythm 2021<br><br>Songnan W, Shengma C. GW24-e2483 Catheter ablation of atrial fibrillation in patients with autoimmune rheumatic diseases. Heart 2013;99(Suppl 3):A197-A97.<br><br>Giallafos I, Triposkiadis F, Oikonomou E, et al. Incident
	Rheumatoid arthritis	
	Spondyloarthropathies	
	Vasculitides	

		atrial fibrillation in systemic sclerosis: the predictive role of B-type natriuretic peptide. Hellenic J Cardiol 2014;55:313-21.<br><br>Pugnet G, Gouya H, Puéchal X, et al. Cardiac involvement in granulomatosis with polyangiitis: a magnetic resonance imaging study of 31 consecutive patients. Rheumatology 2017;56(6):947-56.<br><br>Lindhardsen J, Ahlehoff O, Gislason GH, et al. Risk of atrial fibrillation and stroke in rheumatoid arthritis: Danish nationwide cohort study. BMJ 2012;344<br><br>Each of the subtypes of rheumatological disease are associated with differing risks of development of AF. Here they have been categorised in clinical sub-type.
Smoking	Current	See table S4
	Ex	
Surgery (non-cardiac)	Colorectal	See table S4
	Thoracic	
	Vascular	
Systemic Embolism	-	-
Valvular heart disease	Mitral stenosis / rheumatic valvular disease	See table S4
	Non-mitral valve / other valves	
	Mitral regurgitation	
Vascular dementia	-	-

Supplementary Results

Supplement Table S7. Baseline characteristics of training and testing datasets

	**Training set**<br>**n (%)**	**Testing set**<br>**n (%)**
	1 664 911	416 228
Demographics		
Age, years	49.90 (15.43)	49.90 (15.42)
Sex (women)	844 083 (50.7)	211 478 (50.8)
Comorbidities		
Diabetes mellitus	58 513 (3.5)	14 268 (3.4)
Stroke or TIA	30 871 (1.9)	7 794 (1.9)
Ischaemic heart disease	62 980 (3.8)	15 622 (3.8)
Hypertension	200 217 (12.0)	50 106 (12.0)
Heart failure	11 577 (0.7)	2 790 (0.7)
Dyslipidaemia	48 719 (2.9)	12 170 (2.9)
Hyperthyroidism	13 069 (0.8)	3 233 (0.8)
COPD	20 294 (1.2)	5 129 (1.2)
Chronic kidney disease	23 794 (1.4)	6 014 (1.4)
Anaemia	53 962 (3.2)	13 383 (3.2)
Cancer	58 725 (3.5)	14 783 (3.6)
Valvular heart disease	7 946 (0.5)	1 927 (0.5)
Mean CHA_2DS_2-VASc score	0.98 (1.04)	0.98 (1.04)

Nadarajah R, *et al. Heart* 2023; 109:1072–1079. doi: 10.1136/heartjnl-2022-322076

Supplementary Table S8. Net reclassification using FIND-AF

AF cases

	**FIND-AF**			**FIND-AF**	
**CHA_2DS_2-VASc**	≥0.4%	<0.4%	**C_2HEST**	≥0.4%	<0.4%
≥0.4%	1 121	37	≥0.4%	893	10
<0.4%	82	191	<0.4%	310	218

(green)	Appropriate upclassification
(red)	Inappropriate downclassification

Non-AF cases

	**FIND-AF**			**FIND-AF**	
**CHA_2DS_2-VASc**	≥0.4%	<0.4%	**C_2HEST**	≥0.4%	<0.4%
≥0.4%	65 322	17 511	≥0.4%	38 640	3 053
<0.4%	16 417	315 547	<0.4%	43 099	330 005

(green)	Appropriate downclassification
(red)	Inappropriate upclassification

Net reclassification indices

Index	**CHA_2DS_2-VASc**	**C_2HEST**
Case reclassification (NRI+ [95% CI])	0.031 (0.026-0.048)	0.021 (0.19-0.23)
Non-case reclassification (NRI- [95% CI])	0.0026 (0.0015-0.0032)	-0.096 (-0.098 - -0.095)
Net reclassification (NRI [95% CI])	0.032 (0.029-0.051)	0.113 (0.098-0.135)

Nadarajah R, *et al. Heart* 2023; 109:1072–1079. doi: 10.1136/heartjnl-2022-322076

Supplementary Table S9. Baseline characteristics of testing set, stratified by incident AF and predicted AF risk

	**Incident atrial fibrillation**		**FIND-AF predicted risk**	
	**no AF** **n (%)**	**AF** **n (%)**	**Lower risk** **n (%)**	**Higher risk** **n (%)**
	414 676	1 552	333 286	82 942
Demographics				
Age, years	49.82 (15.38)	73.87 (12.47)	44.11 (10.40)	73.24 (8.75)
Sex (women)	210 646 (50.8)	755 (48.6)	170 568 (51.2)	41 210 (49.7)
Ethnicity				
Asian	8 258 (2.0)	21 (1.5)	7 385 (2.2)	894 (1.1)
Black	6 390 (1.5)	9 (0.6)	5 786 (1.7)	613 (0.7)
Other	27 805 (6.7)	106 (7.4)	22 033 (6.6)	5 878 (7.1)
Unknown	93 630 (22.6)	36 (2.5)	91 505 (27.5)	2 161 (2.6)
White	278 714 (67.2)	1 259 (88.0)	206 577 (62.0)	73 396 (88.5)
Comorbidities				
Diabetes mellitus	14 649 (3.5)	171 (11.0)	6 328 (1.9)	8 072 (9.7)
Stroke or TIA	7 467 (1.8)	189 (12.2)	1 376 (0.4)	6 375 (7.7)
Ischaemic heart disease	15 483 (3.7)	314 (20.2)	3 299 (1.0)	12 486 (15.1)
Hypertension	49 494 (11.9)	621 (40.0)	20 139 (6.0)	29 594 (35.7)
Heart failure	2 745 (0.7)	132 (8.5)	163 (0.0)	2 748 (3.3)
Dyslipidaemia	12 122 (2.9)	121 (7.8)	6 095 (1.8)	5 984 (7.2)
Hyperthyroidism	3 203 (0.8)	44 (2.8)	1 883 (0.6)	1 370 (1.7)
COPD	4 987 (1.2)	106 (6.8)	1 111 (0.3)	4 019 (4.8)
Chronic kidney disease	5 839 (1.4)	99 (6.4)	2 938 (0.9)	2 990 (3.6)

Nadarajah R, *et al. Heart* 2023; 109:1072–1079. doi: 10.1136/heartjnl-2022-322076

Anaemia	13 165 (3.2)	106 (6.8)	9118 (2.7)	4251 (5.1)
Cancer	14 710 (3.5)	186 (12.0)	6120 (1.8)	8303 (10.0)
Valvular heart disease	1 881 (0.5)	84 (5.4)	562 (0.2)	1414 (1.7)
Mean CHA_2DS_2-VASc score (SD)	0.97 (1.03)	2.74 (1.40)	0.62 (0.62)	2.42 (1.14)

AF, atrial fibrillation; CHA_2DS_2-VASc, Congestive heart failure, Hypertension, Age >75 years [2 points], Stroke/transient ischemic attack/thromboembolism [2 points], Vascular disease, Age 65-74 years, Sex Category; COPD, chronic obstructive pulmonary disease; TIA, transient ischaemic attack

Nadarajah R, *et al. Heart* 2023; 109:1072–1079. doi: 10.1136/heartjnl-2022-322076

Supplement Figure S1. Calibration plots

FIND-AF

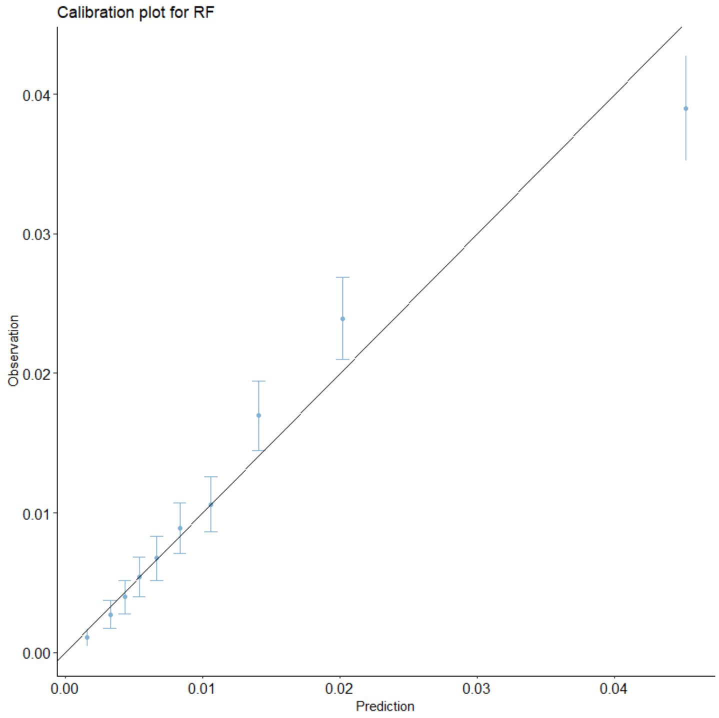

Multivariable logistic regression

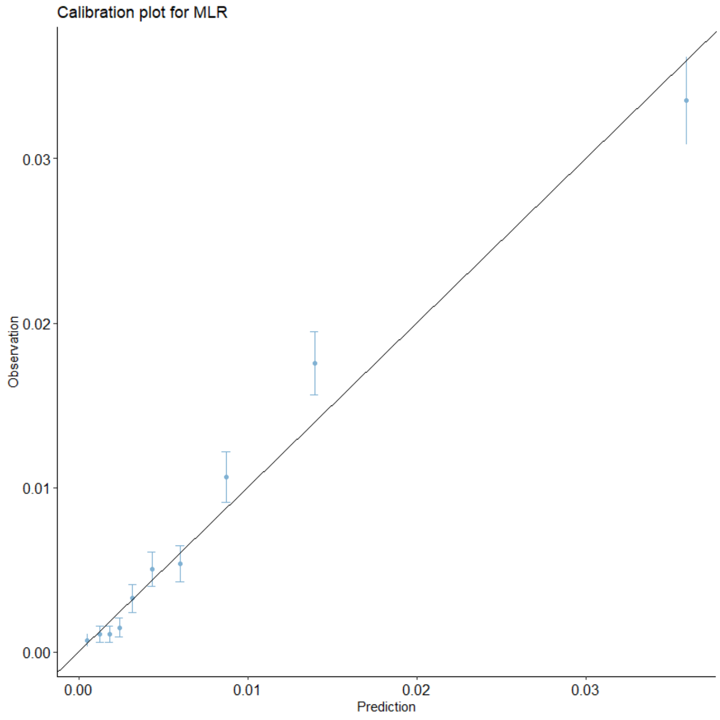

CHA_2DS_2VASc

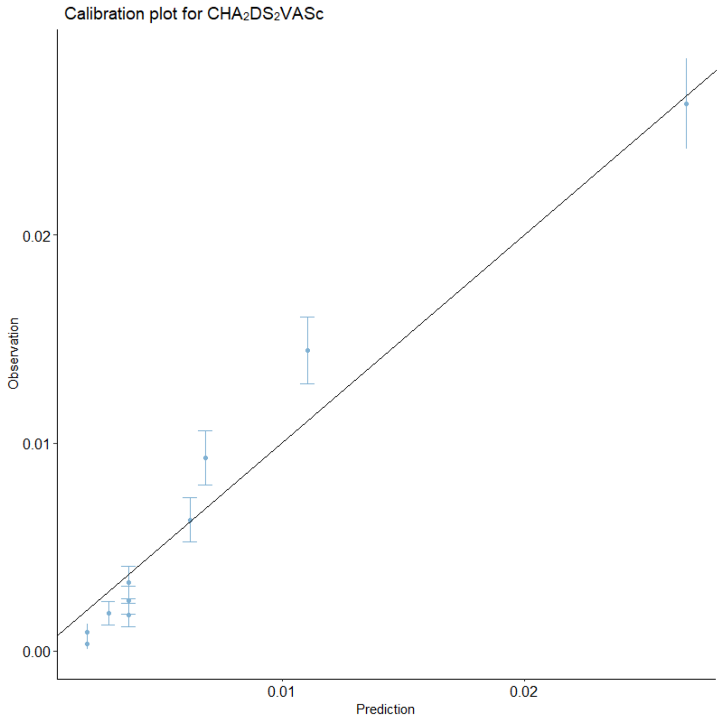

C_2HEST

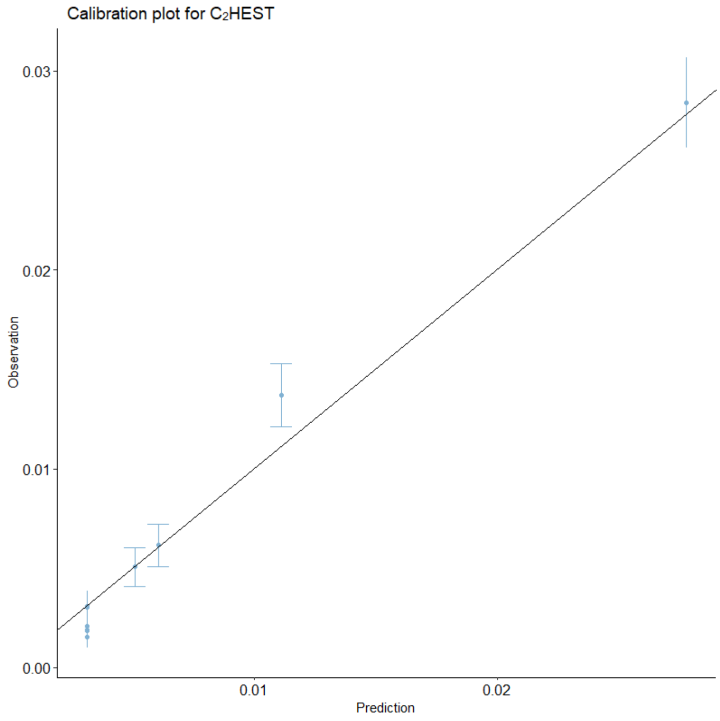

Supplementary Figure S2. Decision curve analysis for FIND-AF versus CHA_2DS_2-VASc and C_2HEST

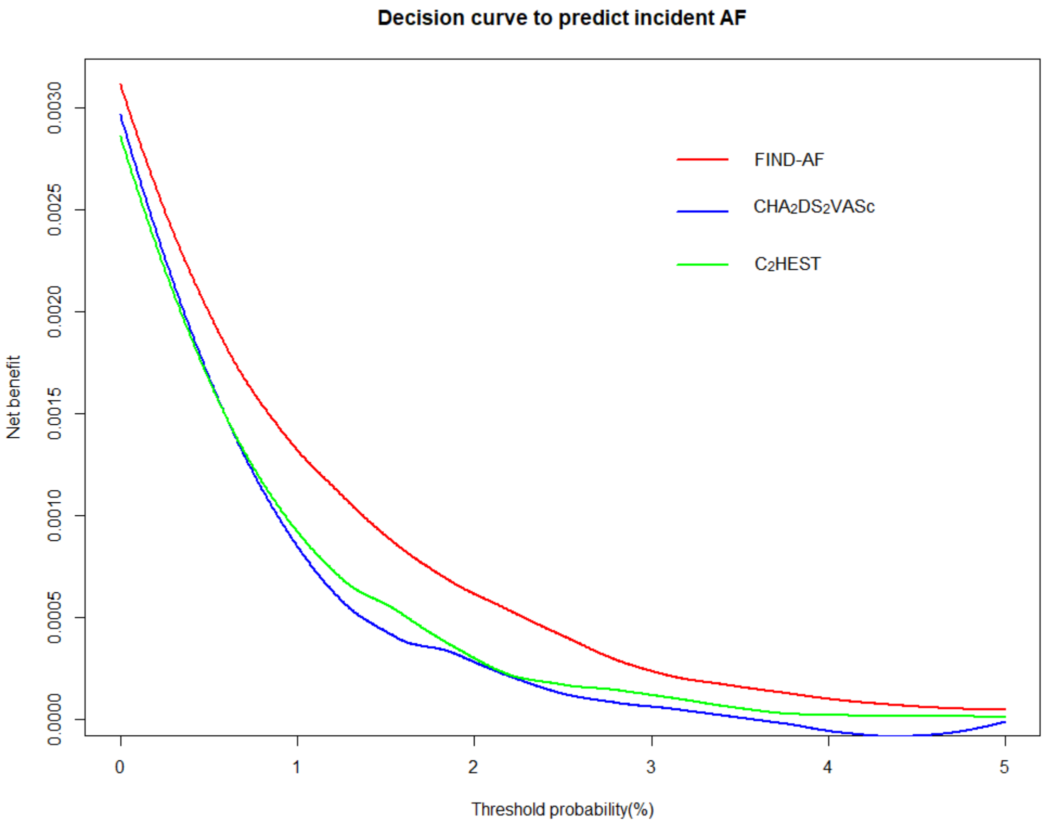

Nadarajah R, *et al. Heart* 2023; 109:1072–1079. doi: 10.1136/heartjnl-2022-322076

References

1. Raileanu LE, Stoffel K. Theoretical comparison between the gini index and information gain criteria. *Annals of Mathematics and Artificial Intelligence* 2004;41(1):77-93.
